# Prevention and treatment of sepsis-induced acute kidney injury: an update

**DOI:** 10.1186/s13613-015-0095-3

**Published:** 2015-12-21

**Authors:** Patrick M. Honore, Rita Jacobs, Inne Hendrickx, Sean M. Bagshaw, Olivier Joannes-Boyau, Willem Boer, Elisabeth De Waele, Viola Van Gorp, Herbert D. Spapen

**Affiliations:** Intensive Care Department, Universitair Ziekenhuis Brussel, Vrije Universiteit Brussel, Laarbeeklaan 101, 1090 Brussels, Belgium; Division of Critical Care Medicine, Faculty of Medicine and Dentistry, University of Alberta, Edmonton, AB Canada; Haut Leveque University Hospital of Bordeaux, University of Bordeaux 2, Pessac, France; Department of Anaesthesiology and Critical Care Medicine, Ziekenhuis Oost-Limburg, Genk, Belgium

**Keywords:** Sepsis, Acute kidney injury, Septic acute kidney injury, Prevention, Treatment, Review

## Abstract

Sepsis-induced acute kidney injury (SAKI) remains an important challenge in critical care medicine. We reviewed current available evidence on prevention and treatment of SAKI with focus on some recent advances and developments. Prevention of SAKI starts with early and ample fluid resuscitation preferentially with crystalloid solutions. Balanced crystalloids have no proven superior benefit. Renal function can be evaluated by measuring lactate clearance rate, renal Doppler, or central venous oxygenation monitoring. Assuring sufficiently high central venous oxygenation most optimally prevents SAKI, especially in the post-operative setting, whereas lactate clearance better assesses mortality risk when SAKI is present. Although the adverse effects of an excessive “kidney afterload” are increasingly recognized, there is actually no consensus regarding an optimal central venous pressure. Noradrenaline is the vasopressor of choice for preventing SAKI. Intra-abdominal hypertension, a potent trigger of AKI in post-operative and trauma patients, should not be neglected in sepsis. Early renal replacement therapy (RRT) is recommended in fluid-overloaded patients’ refractory to diuretics but compelling evidence about its usefulness is still lacking. Continuous RRT (CRRT) is advocated, though not sustained by convincing data, as the preferred modality in hemodynamically unstable SAKI. Diuretics should be avoided in the absence of hypervolemia. Antimicrobial dosing during CRRT needs to be thoroughly reconsidered to assure adequate infection control.

## Background

Both sepsis and acute kidney injury (AKI) are diseases of major concern in critically ill patients. Severe sepsis is often complicated by AKI [1–4]. The overall incidence of septic AKI (SAKI) among all intensive care unit (ICU) admissions ranges between 15 and 20 % [2]. Large studies in critically ill patients convincingly demonstrated the “intimate” bond between AKI and sepsis. For instance, the BEST Kidney and FINNAKI studies, which covered different time periods, both reported AKI in up to half of the septic patients [3, 4]. In a large analysis of 14,039 SAKI patients from ICUs in Australia and New Zealand, the proportions of patients stratified for risk, injury, and failure according to the RIFLE criteria were 38.5, 38.8, and 22.7 %, respectively [1]. Medical admissions necessitating mechanical ventilation and/or with a long ICU stay were at the highest risk. SAKI highly determines ICU outcome [1]. The BEST Kidney investigators reported a 70 % overall hospital mortality in patients with SAKI [3]. Prognosis worsened with increasing age and severity of illness, use of vasoactive drugs, and mechanical ventilation [3]. In contrast, an Indian study reported an overall mortality of 52 % which was directly correlated with age, disease severity, and degree of non-renal organ failure [5]. The recent IVOIRE study showed a similar mortality of around 50 % at 90 days in SAKI patients with a cardiovascular SOFA score of 3–4 and under CRRT [6]. This apparent decrease of mortality over time probably implies a more adequate management of SAKI [7]. We review some important developments in prevention and treatment of SAKI which have contributed to this improved prognosis or hold promise for further amelioration.

## Pathophysiology of SAKI

The pathophysiology of SAKI is much more complex than previously anticipated [8]. Primary conditions associated with AKI, such as sepsis, major surgery, heart failure, and hypovolemia, may all be complicated by shock. Thus, it is tempting to attribute AKI to ischemia and systemic hemodynamic changes [9]. However, renal dysfunction does not result from hypoperfusion alone but may emanate to a large extent from renal inflammation and tubular responses to various sepsis mediators. In many patients, AKI occurs without overt signs of global renal hypoperfusion and SAKI has been described in the presence of normal or even increased renal blood flow [9]. This explains why the sole correction of hemodynamic parameters often fails to prevent SAKI [8, 9]. Taken together, the pathophysiology of SAKI is no longer based on an ischemia/reperfusion paradigm but rather embraces an aggregate of inflammation, microcirculatory dysfunction, perfusion deficit, bio-energetic reactions, and tubular cell adaptation to injury [9]. It must be admitted, however, that an intrinsic role of these pathophysiological “subsets” underlying SAKI has been derived more from hypothesis-generating experience than from concordant state-of-knowledge.

## Prevention of sepsis-induced AKI

### Fluid resuscitation

#### Aim

The old “credo” stating that fluid harms the lung but benefits the kidney should be revised [10]. Liberal fluid administration is of key importance to optimize systemic hemodynamics in patients with SAKI. Yet, ongoing controversy exists about efficacy, nature, extent and duration of fluid therapy in septic shock [11]. In fact, ICU physicians are faced with a “double-edged” fluid dilemma. Volume resuscitation is indeed essential to restore and maintain cardiac output and oxygen delivery. Sustained or unrestricted infusion of fluids, however, will cause tissue edema which significantly contributes to organ dysfunction. On the other hand, too rapid or excessive fluid removal with diuretics or extracorporeal techniques may expose patients to severe hypovolemia and recurrent renal injury. An optimal fluid management would be to guarantee a stepwise and smooth transition from initial unrestricted fluid administration (positive fluid balance) over a state of equilibrium (steady-state fluid balance) to appropriate fluid removal (negative fluid balance) [12]. This process is kept on track by meticulous serial assessments of fluid handling aiming at well-defined cardiovascular and renal targets. Low intravascular oncotic pressure—the typical hallmark of patients with systemic inflammatory response syndrome (SIRS) and septic patients without AKI—is also observed in patients who develop SAKI [13, 14]. This explains their particular vulnerability to potentially harmful fluid accumulation as compared with non-septic AKI patients [13]. Moreover, renal ischemia and reperfusion is associated with reduced capillary blood flow and loss of glycocalyx integrity [14]. Early aggressive fluid resuscitation can be life-saving [15], yet several observational studies in critically ill patients with SAKI have linked fluid overload to increased mortality and reduced kidney recovery [16, 17]. RRT may provide better control of fluid balance in this population. However, a mortality benefit of RRT is unproven and timing and dose remain matter of debate.

#### Type of fluid

##### Crystalloids versus colloids

Hypotension and hypovolemia during sepsis may cause or worsen AKI. Evidence is accumulating suggesting that crystalloid but not colloid solutions should be used for initial intravascular volume expansion in septic patients at risk for AKI [18–20]. The substantial risk for induction of osmotic nephrosis (by pinocytosis in the renal tubules) strongly pleads against the use of hydroxyethyl starch and dextran solutions [18–22].

##### Balanced crystalloids versus isotonic salt solutions

Balanced crystalloid perfusions (e.g., Ringer’s lactate, Plasmalyte^®^) are associated with less occurrence of AKI than isotonic salt solutions [23]. The latter contain a too high chloride load which is thought to be detrimental for the kidney by inducing vasoconstriction in the renal vascular bed [23–25] and was independently associated with increased morbidity and mortality [22, 26]. Reported differences in incidence of AKI between patients receiving either buffered or saline crystalloids may also be influenced by unidentified confounding factors [27, 28]. Two recent retrospective trials in patients with sepsis [29] and SIRS [30] divulged an association between infusion of normal saline and increased in-hospital mortality. However, a recent large prospective randomized trial (Split Trial) reported no difference in AKI incidence in patients receiving either balanced crystalloids or isotonic salt solutions [31]. Thus, any suggested superiority of balanced crystalloids to isotonic saline for preventing SAKI remains to be proven.

#### Albumin

It is common belief that albumin solutions do force excess tissue water back into the endovascular space by creating a hyperoncotic effect. Surprisingly, this has never been evidenced [32]! Albumin infusion may even promote extracellular fluid overload without improving hypovolemia in sepsis complicating advanced cirrhosis or diabetes [33]. Any beneficial effect of albumin on patient outcome remains controversial. Data from the SAFE study [34] and a systematic review [35] showed that the use of albumin-containing solutions for the resuscitation of patients with sepsis was associated with lower mortality and did not impair renal function compared with other fluid resuscitation regimens. The recently published ALBIOS trial failed to show a mortality benefit in patients with severe sepsis who were fluid resuscitated with albumin and crystalloids as compared with crystalloids alone [36]. However, a post hoc analysis showed a significantly lower 90-day mortality in a subgroup of 1121 patients with septic shock treated with albumin [36]. Moreover, a recent review highlighted that ALBIOS patients who received albumin needed less vasopressive support and achieved a significantly better fluid balance [37]. Still, many uncertainties prevail as to the potential benefit, indications, and cost-effectiveness of albumin [38, 39].

#### Early use of continuous RRT (CRRT)

Fluid overload definitely increases kidney edema and enhances severity and irreversibility of SAKI [15–17]. Therefore, timely use of CRRT in case of fluid overload that is poorly responding or refractory to diuretics might be a reasonable approach to attenuate or control SAKI [40]. Weight gain at initiation of RRT has been associated with a poor outcome [41]. Awaiting further trials, the early use of CRRT is a reasonable yet not generally accepted approach to control fluid homeostasis.

### Monitoring

#### Electronic AKI “sniffers”

Real-time electronic reporting systems have been developed to help recognizing AKI at an earlier stage [42] and to determine the eventual need for RRT [43, 44]. An AKI alert is based on either RIFLE [42, 44] or AKIN [43] criteria. Ideally, AKI alert systems should be based on kidney improving global outcomes (KDIGO) criteria [45]. Although preliminary results were promising, a recent trial using these criteria did not confirm that early electronic detection of AKI improved outcome [46].

#### Renal Doppler

The kidneys receive approximately 25 % of the total blood flow. Yet, they only use half of this flow mainly because of intricate intra-renal shunting [47]. Monitoring of global renal blood flow thus provides little information about the adequacy of oxygen supply to the kidneys [47]. As a consequence, renal Doppler is not a reliable tool to assess renal oxygen supply and its eventual response to fluid loading [48]. Future research rather should focus on the renal microcirculation. A pilot study of Schneider et al. used contrast-enhanced ultrasound (CEUS) to evaluate renal cortical perfusion in elective cardiac surgery patients. CEUS was feasible, well tolerated and results were reproducible. CEUS-derived parameters suggested a decrease in renal perfusion within 24 h of surgery [49] which persisted after correction of hemoglobin [49].However, recently reported experience with CEUS was disappointing with regard to clinico-radiological correlation and reproducibility [50, 51]. In addition, CEUS may have questionable accuracy in the presence of important intra-renal and eventual peri-glomerular shunting [52].

#### Central venous oxygen saturation (S_cv_O_2)_ and lactate clearance rate

##### S_cv_O_2_

Boosting systemic oxygen delivery under guidance of S_cv_O_2_ has recently been shown to prevent or avoid progression of AKI but had no effect on mortality [53]. Recent studies, however, did not find a correlation between S_cv_O2 values and AKI incidence [54]. Kidney performance is less influenced by enhanced oxygen delivery but strongly depends on adequate arterial perfusion pressure [55]. This explains why noradrenaline better preserves kidney function than dobutamine [56]. Setting higher S_cv_O_2_ targets is an attractive approach for preventing SAKI [57] but more robust data regarding its feasibility and effectiveness in clinical practice are awaited.

##### Lactate clearance rate

Lactate levels more appropriately reflect arterial perfusion than oxygen supply, especially when accounting for the high level of intra-renal oxygen shunting [47]. Lactate clearance rate could thus mirror kidney perfusion more adequately than S_cv_O_2_ [47]. Lactate is a powerful predictor of mortality in patients with SAKI and improving lactate clearance has been associated with a better outcome [57]. Janssen et al. reported that lactate-guided therapy reduced the incidence of AKI in ICU patients [58]. In contrast, Jones et al. found that additional targeting of normal lactate levels as compared with aiming at higher S_cv_O_2_ did not influence in-hospital mortality in septic shock patients resuscitated to normal central venous and mean arterial pressure (MAP) [59]. More outcome data from prospective lactate-driven resuscitation protocols are eagerly awaited.

#### Central venous pressure (CVP) and kidney “afterload”

For decades, clinicians estimated that preload was the main determinant of kidney function. Increasing preload was thought to increase renal blood volume and flow. Although a “critical” CVP level is required to ensure optimal renal function, unrestrained preload increase may harm the kidneys by enhancing venous congestion and blocking venous outflow (i.e., increased “afterload”). Recently, higher CVP levels were found to be associated with an increased incidence and morbidity of AKI during septic shock [54]. Relatively small increases in thoracic pressure already compromised venous return to the kidneys [54]. The kidneys are encapsulated and thus extremely vulnerable to compression by evolving edema. AKI due to such “kidney compartment syndrome” may be an early sign of abdominal hypertension [60]. CVP is a poor indicator of renal perfusion and, if used, serial readings are more useful than isolated values. An optimal CVP level is unknown but unrestrained “pumping up” of preload as protective measure for SAKI is obsolete [54, 55, 60].

### Differentiating transient (functional) SAKI from structural SAKI

#### Urine biochemistry

SAKI may present either as a functional or structural entity. The difference is clinically relevant since functional SAKI can be reversed completely by early adequate treatment whereas structural kidney damage will mostly require RRT. Discriminating functional from structural SAKI at the bedside, however, remains challenging. Low fractional excretions of sodium (FE_Na_) and urea (FE_Urea_) are highly prevalent during the initial phase of sepsis. Oliguria is an earlier sign of impending SAKI than the increase in serum creatinine. It is assumed that high FE_Na_ and low FE_Urea_ values are associated with intrinsic SAKI whereas high values of both FE_Na_ and FE_Urea_ concur with transient or functional SAKI. However, a definite discriminative power of these urinary indices has not been established [61]. They are also less specific than currently tested biomarkers of SAKI [62] and less accurate for differentiating transient from persistent AKI [63] and SAKI from non-SAKI [64].

#### Biomarkers

Among various biomarker assays, neutrophil gelatinase-associated lipocalin, urine insulin-like growth factor-binding protein 7, and tissue inhibitor of metalloproteinases-2 are the most promising [65, 66]. Still, these markers have limited availability and thus cannot be advocated for routine guidance of therapy. Bagshaw et al. prospectively showed that urinary sodium, FE_Na_, and FE_Urea_ did not reliably predict biomarker release, worsening of AKI, need for RRT or mortality [64].

#### Oliguria vs creatinine

Oliguria is an earlier sign of impending SAKI than the increase in serum creatinine [61]. Macedo et al. reported that oliguric episodes occurred frequently in ICU patients and allowed to identify more AKI as compared to serum creatinine [67]. In contrast, other investigators found a poor specificity of oliguria [68, 69]. To date, no biological or laboratory marker can be put forward that reliably distinguishes functional from structural SAKI.

### Transfusion policy

An optimal hematocrit level may contribute to SAKI prevention [70] but this is not supported by clinical data. A hematocrit value below 24 % was associated with significantly more post-operative AKI in cardiac surgery patients with systemic inflammatory response syndrome who are known to display a sepsis-alike inflammatory state [71]. Whether a similar target should be pursued in patients with SAKI remains to be proven. A recent retrospective study showed that red blood cell (RBC) transfusion in non-bleeding critically ill patients with moderate anemia and without shock was associated with higher nosocomial infection rates, more AKI, and increased mortality [72]. This apparent “transfusion-related AKI” could be coined by the acronym “TRAKI” in analogy to “TRALI” which stands for “transfusion-related acute lung injury” [72, 73]. As in TRALI, TRAKI may result from endothelial injury [74] as most of the protective glycocalyx layer above the endothelium is damaged and lost during severe sepsis [14]. Of note is that the introduction of citrate as an anticoagulant for CRRT resulted in significantly lower transfusion needs. A potential beneficial role of citrate in prevention and/or recovery of SAKI has been suggested [75] but needs confirmation. The FINNAKI study showed that the age of transfused RBCs was independently associated with in-hospital mortality but not 90-day mortality or KDIGO stage 3 AKI [76].

### Vasopressive and inotropic support

As discussed earlier, a decreased renal blood flow and oxygen supply were wrongly assumed to be the main instigators of SAKI [8, 77]. This directed treatment towards increasing filling pressures (i.e., fluid administration) and/or cardiac output (i.e., inotropic support). In an experimental hypotensive and hyperdynamic septic shock model, Di Giantomasso et al. demonstrated that vasopression with noradrenaline significantly increased global and medullary renal blood flow and restored normal renal vascular tone [78]. In the same model, angiotensin II infusion decreased renal blood flow while markedly increasing diuresis and normalizing creatinine clearance [79]. Low-dose vasopressin did not reduce mortality rates as compared with noradrenaline among patients with septic shock [80]. In patients with SAKI, vasopressin only reduced progression to stage I but not to more severe AKI stages [81]. An optimal perfusion pressure has not yet been determined. Asfar et al. proposed (in a randomized controlled trial) a MAP of 65–70 mmHg as reasonable objective [82] whilst another (observational) study found that MAP values between 72 and 82 mmHg were necessary to prevent AKI in patients with septic shock and initially impaired renal function [83].

### Intra-abdominal hypertension

Intra-abdominal hypertension (IAH) and its most dreaded presentation, the abdominal compartment syndrome (ACS), are frequently associated with AKI in surgical and trauma patients. Because signs and symptoms are non-specific and laboratory and imaging studies often remain inconclusive, the diagnosis of AKI as a manifestation of IAH requires a high index of clinical suspicion. Early recognition and treatment improve clinical outcome [84]. IAH has also been described in up to one-third of cardiac surgery patients where it was found to be strongly associated with higher baseline intra-abdominal pressure (IAP), increased CVP, positive fluid balance, extracorporeal circulation, use of vasoactive drugs and AKI. Determinants of IAH need accurate assessment and patients with any known risk factor(s) must be closely monitored during the perioperative period. In this context, the baseline IAP may be a valuable early warning parameter for IAH [85]. According to the most recent guidelines [86], IAP should be monitored in all surgical patients at risk for AKI (i.e., cardiac surgery, complicated abdominal surgery and post-operative sepsis) [84–86]. IAP is best measured with a bladder catheter. No consensus exists on whether AKI can be prevented by early abdominal decompression or administration of diuretics [84–86]. RRT may facilitate or improve volume management in some cases but cannot be recommended as standard treatment [84–86]. IAH and ACS also increase morbidity and mortality in medical ICU patients. Factors predisposing to IAH/ACS in this population include sepsis, large-volume fluid resuscitation, polytransfusion, mechanical ventilation with high intrathoracic pressures, and acidosis.

## Treatment of sepsis-induced AKI

### Dosing of CRRT

High-volume hemofiltration offers no mortality benefit in SAKI [6, 87, 88]. Based on two large seminal trials with an important septic subpopulation [89, 90], a CRRT dose of 20–25 ml/kg/h is currently issued by the KDIGO guidelines [45]. The prescribed dose should be somewhat higher to ascertain delivery of at least 20–25 ml/kg/h [91]. A 25–30 ml/kg/h dose may be more convenient in sepsis but no strong data support its recommendation [92].

### Timing of (C) RRT

(C)RRT should be initiated when fluid overload is poorly tolerated and only partly or not responsive to a diuretic challenge [15]. The IVOIRE study demonstrated that starting CRRT at RIFLE injury level in established SAKI was associated with a very low 90-day mortality [87]. ICU patients requiring RRT, however, showed marked variation in factors that influence start of RRT. RRT initiation with fewer clinical triggers was associated with lower mortality [93]. In the recent observational study from the FINNAKI group, applying RRT for conventional indications at an early stage was also associated with lower mortality [94]. Timing of RRT may modify survival but awaits appraisal in three forthcoming randomized trials [95–97]. Meanwhile, it is acceptable to start CRRT at RIFLE injury/failure level as dictated by the KDIGO guidelines [45].

### Renal replacement modalities

Prowle and Bellomo reported that hemodynamically unstable patients with SAKI treated with CRRT remained significantly less dialysis-dependent than those receiving intermittent hemodialysis (IHD) [98]. A recent meta-analysis by Schneider also suggested that CRRT outperformed IHD in obtaining renal recovery in patients without cardiovascular instability [99]. This meta-analysis is prone to criticism because many of the included studies are old, uncontrolled, or compare CRRT with inappropriately managed IHD [99]. Continuous veno-venous hemofiltration (CVVH) was associated with a trend towards early reduction of vasopressor support [100]. To date, however, there are no strong decisive arguments to prefer CRRT or IHD as primary treatment for SAKI, except maybe in severely hemodynamically unstable patients (level 2B in the KDIGO guidelines) [45]. A large and sufficiently powered randomized trial comparing the effect of CRRT vs. IHD on renal recovery as primary endpoint would end this controversy.

### Diuretics

The use of diuretics to induce or increase urine production in the absence of hypervolemia is associated with increased mortality [101] and should be discouraged. In contrast, diuretics might improve outcome when fluid balance remains positive or in case of overt fluid overload [102]. However, any beneficial effect of diuretics on mortality was lost after adjustment for fluid balance [102]. Ho and Power reviewed the use of furosemide in AKI and found no beneficial effects on mortality [103]. A furosemide stress test for early assessment of tubular function showed robust capacity to identify patients at risk for severe and progressive AKI [104] but needs validation in SAKI.

### Antimicrobial dosing during CRRT

CRRT significantly influences the pharmacokinetic and pharmacodynamic behavior of most antimicrobial agents. This is insufficiently anticipated by currently recommended dosing guidelines. Patients are particularly at risk for underdosing which may cause treatment failure and enhanced resistance. An in-depth discussion of antimicrobial handling during CRRT is beyond the scope of this review. Table 1 summarizes literature-based [105–112] dose recommendations for some major antibiotic and antifungal drugs during CVVH at a dose of 25 ml/kg/h. Colistin deserves special attention. Our group has shown that patients undergoing CVVH can support long-term colistin therapy at doses up to 4.5 million IU tid [108]. CVVH thus may act as a “shield” providing sufficiently high plasma colistin levels whilst avoiding toxicity [113]. Importantly, safe application of such treatment requires the use of filter membranes that allow high colistin adsorption in association with citrate anticoagulation to preserve optimal convective elimination capacity [114].

**Table 1:** Dose recommendations for some frequently used antimicrobials during CRRT, (CVVH mode; 25 ml/kg/h)

Antimicrobial	Loading dose	Maintenance dose
Amikacin	30–35 mg/kg	TDM
Meropenem	2 g	2 g over 3 h tid
Piperacillin-tazobactam	4 g/0.5 g	16 g/2 g (CI)
Vancomycin	35 mg/kg over 4 h	30 mg/kg (TDM = 25–30 mg/L)
Teicoplanin	15 mg/kg bid	600 mg od
Linezolid		600 mg tid
Ciprofloxacin	800 mg	400 mg tid
Tigecycline	150 mg	100 mg bid
Colistin	9 MIU	4,5 MIU tid
Voriconazole	8 mg/kg bid	6 mg/kg bid
Fluconazole		600 mg bid
Cefepime		2 g tid
Gentamycin		7 mg/kg od
Bactrim	1200 mg/240 mg (3amp)	800 mg/160 mg (2amp) tid
Clindamycin		900 mg qid

Adapted from references [105–113]

*TDM* therapeutic drug monitoring, *od* once daily, *bid* twice daily, *tid* three times daily, *qid* four times daily, *amp* ampules, *CI* continuous infusion, *MIU* million units

### Other blood purification strategies

Several strategies are under investigation including newly designed membranes [114], apheresis or selective plasma exchange [115] and polymyxin B hemoperfusion [116]. These purification strategies fit in the concept of host inflammatory response modulation, yet have not proven successful [117]. Hyperadsorptive membranes such as the acrylonitrile 69 surface-treated or polymethylmethacrylate filters very effectively adsorb crucial inflammatory mediators (e.g., high-mobility group box 1 protein (HMGB-1)) [118]. Although small-sized (26 kDa), HMGB-1 was not removed by convection but entirely by adsorption [118]. A preliminary study of polymyxin B hemoperfusion added to conventional therapy showed significantly improved hemodynamics, less organ dysfunction and reduced 28-day mortality in patients with severe sepsis and/or septic shock from abdominal origin [116]. However, a recently published multicenter randomized controlled study demonstrated a non-significant increase in mortality and no improvement in organ failure with polymyxin B hemoperfusion compared to conventional treatment of peritonitis-induced septic shock [119].

### Medical therapies

A small randomized phase I trial showed that adjunctive treatment with recombinant alkaline phosphatase (RAP) could prevent SAKI and even reversed some cases of advanced SAKI [120]. A phase II clinical trial is currently ongoing [121]. The exact mechanism and optimal timing of action of RAP is still unclear. RAP probably combats renal inflammation through dephosphorylation of lipopolysaccharide and adenosine triphosphate [122, 123].

## Conclusions

Prevention of SAKI starts with early and adequate fluid resuscitation. Crystalloids are preferred over colloids but balanced crystalloids do not appear superior to classic crystalloids for counteracting SAKI. Synthetic colloids and starches in particular should be withheld. No data support the use of albumin in patients with SAKI. Regarding SAKI prevention, S_cv_O_2_ monitoring performs better than lactate clearance rate or renal Doppler for monitoring kidney perfusion. Lactate clearance rate, however, is better correlated with SAKI-related mortality. High filling pressures must be avoided in light of the detrimental effects imposed by an increased kidney “afterload”. Noradrenaline remains the vasopressor of choice for preventing SAKI. Vasopressin or analogs need further investigation. IAH is a potential, yet often overlooked, trigger of SAKI. Early initiation of RRT is indicated when fluid overload is excessive or refractory to diuretics. CRRT is increasingly considered as first-choice therapy in hemodynamically unstable SAKI. Expanding its use to “stable” SAKI patients is attractive but not supported by current literature. Except for life-threatening hypervolemia, diuretics have no place in prevention or treatment of SAKI. Most antimicrobials require dose adaptation during CRRT.
